# An open-source, battery-powered, low-cost, and dual-channel pneumatic pulse generator for microfluidic cell-stretch assays

**DOI:** 10.1016/j.ohx.2024.e00595

**Published:** 2024-10-11

**Authors:** Samuel Olson, McKenna Finley, Raviraj Thakur

**Affiliations:** Cancer Early Detection Advanced Research Center (CEDAR), Knight Cancer Institute, Oregon Health & Science University, USA

**Keywords:** Cell-stretch assays, Microfluidic pressure controller

## Abstract

Cells in the body are regularly subjected to mechanical forces that influence their biological fate in terms of morphology, gene expression, and differentiation. The current gold standard method to replicate these effects in vitro is to culture cells on devices with elastic substrates and to impart mechanical stretch using mechanical or pneumatic pull–push methods. Microfluidic device designs offer several advantages in this context for general uniform and controlled stretching. However, the experimental setups are bulky, not user-friendly, and often involve several components that reside outside of the tissue culture incubator. Given the wide utility of mechanical stimulation in in-vitro research, our aim was to create a turn-key research tool that bioengineers can deploy in their cell-stretch assays, without having to deal with the complexity and nuances of ad hoc experimental setups. Here, we present an open-source, battery-powered, dual-channel cyclic pneumatic pulse generator box that can reside within an incubator and is compatible with custom microfluidic cell stretch devices. Our method depends on generating pressure-vacuum pulses simply using a linear miniature pneumatic air cylinder actuated using a continuous servo motor. To the best our knowledge, this is a first example of a completely battery-powered, standalone system that doesn’t have any peripherals residing out of the incubator. We provide a detailed list of different components as well as the step-by-step assembly process. We validate its performance in a cell stretch assay using a commercially available microfluidic chip. Our results show an acute stimulation of cyclic stretching over 8 h on human umbilical vein endothelial cells (HUVECs) resulted in preferential alignment of cells perpendicular to the axis of stretch.

## Graphical abstract

**Figure:**
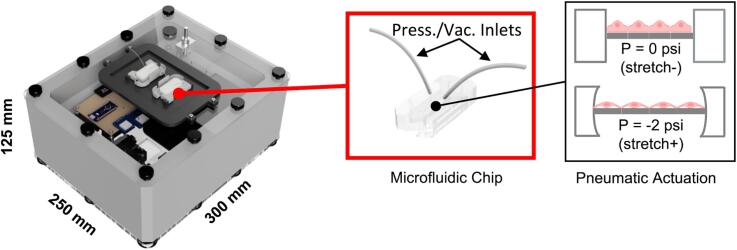

**Specifications table:** 

Hardware name	A pneumatic pressure controller
Subject area	Biological sciences, tissue culture
Hardware type	An in-vitro cell stretching apparatus.
Closest commercial analog	Emulate, Cytostetcher (Curi Bio), ST-1400 and ST-1440 (STREX)
Open source license	This work is licensed under a Creative Commons Attribution 4.0 International License.
Cost of hardware	$350
Source file repository	Mendeley Data: https://doi.org/10.17632/f8jzn29f4c.2

## 1 Hardware in context

Mechanical stress is experienced by cells in various parts of the human body, including the circulatory and digestive systems [1], [2], [3], [4], [5]. These stresses play an important role in various stages of organogenesis and homeostasis, from cellular alignments to regulating gene expression profiles [6]. Mechanical stretching experienced by cells is known to cause increased rates of cell division [7], upregulation of cytokine release in lung epithelial cells [8], and varying effects on fibroblasts depending on the alignment of the cells with the direction of uniaxial stress [9]. Recently, a study conducted at the single-cell level has provided insight into how mechanical stretching impacts the growth of skin cells [10].

Undoubtedly, the ability to test the effect of mechanical stretching on cells in the laboratory is crucial for mechanistic biology and bioengineering model systems for drug screening. Microfluidic devices offer an effective tool to replicate such physiological effects in vitro in miniature devices. One of the common methods involves seeding of human cells or cell lines on elastic poly-dimethylsiloxane (PDMS) membranes and pulling the membrane to induce mechanical stretch. While several device designs have been presented, a major categorical difference is the experimental apparatus used to impart the stretching of the elastic substrate. A mechanically actuated device typically consists of cells cultured on a thin membrane subjected to tensile stress via physical stretching of the thin PDMS membrane [11], [12], [13]. The mechanical pull is generated using a stepper motor and a lead screw assembly to pull the membrane. This method enables high precision over strain as well as strain rate but requires a complex peripheral control system and consumes a large amount of power. On the other hand, pneumatically actuated devices use air pressure and vacuum to achieve the same effect. Here, the peripheral components are comprised of compressed air cylinders and solenoid valves for cyclic switching. While the precision over induced strain is much lower compared to mechanical actuation, these systems are still equally effective as demonstrated by their widespread utility in cell stretching assays [14], [15], [16].

Despite this progress, one critical issue of pneumatically activated cell stretch devices is that the peripheral components must reside outside of the incubator. Thus, the tubing for the pressure source must be fed through to the device in the interior of the incubator. This is of great concern as it sacrifices the quality of the incubation environment and can potentially lead to bacterial and fungal contamination. Additionally, not all tissue culture CO_2_ incubators have access ports, so the current experimental setups are not general purpose. One such example of a commercially available pneumatic cell stretch device is the Emulate Inc Chip-S1™ microfluidic chip, which consists of two 1 mm wide channels separated by a 50 µm thick membrane containing 7 µm diameter pores. Ports on the sides of the channels allow for introduction of positive (negative) pressure, resulting in compressive (tensile) strain on the membrane.

Through this work, we have developed a battery-powered pneumatic system that is compatible with tissue culture incubators and can be used for acute cell stretch assays. We deployed a miniature, two-way, air cylinder coupled to a continuous DC servo motor via a slider-crank mechanism to reliably generate pressure/vacuum pulses. We deployed this mechanism using a combination of commercially available components, 3D printed mounting brackets and laser cut PMMA sheets. We have packaged this assembly in an enclosure that houses this mechanical assembly as well as control hardware using open-source microcontrollers. We describe in detail the electrical components, mechanical components, and firmware of this system. We begin with a brief overview of the entire system, and then present step-by-step instructions for assembly and use. Additionally, we demonstrate its utility by performing an 8-hour acute stretching of human umbilical vein endothelial cells (HUVECs) seeded in commercially available microfluidic devices. We believe that this open-source platform will offer a valuable tool for bioengineers and biologists to perform cell stretching experiments in a simple and reliable way.

## 2 Hardware description

The hardware described here is a machine that enables life science researchers to perform cell stretch assays by generating pressure-vacuum pulses. We essentially demonstrate how to build this prototype using off-the-shelf components and rapid prototyping parts. One of the major distinctions of this apparatus compared to other commercially available or reported devices is that it is portable, and thus compatible with a variety of tissue culture incubators. Secondly, the entire cost of our prototype is ∼$350, much cheaper than any of the existing commercial solutions. Additionally, while cell-stretching is demonstrated as a potential use case for our machine, it is general-purpose and can be used for a variety of applications such as microfluidic flow control, reagent dispensing or bioprinting. Following are some of the highlights of our pressure generator.

• Able to create pressure/vacuum pulses at 0.5–2.5 Hz and up to ±2 psi.
• Operation time of ∼100 h using 26,000 mAh power bank
• General-purpose and can be repurposed for other applications such as fluid control or dispensing.

### 2.1 Pressure controller overview

The electromechanical system presented here is a mechanical and electrical subsystems. The mechanical subsystem consists of the subassembly of miniature air cylinder, slider-crank assembly and continuous DC servo motor. It is held in place with custom 3D printed brackets and fixtures for reliable operation. We have two such sub-assemblies giving us dual-channel ability. We used an off-the-shelf mini air cylinder (Laize, model-CDJ2B10X30-B) which has stroke of 30 mm and bore diameter of 10 mm. We also used an off-the-shelf DC continuous servo from Parallax (Parallax feedback high speed, model SKU 900-00360). The electrical subsystem consists of a microcontroller for interfacing with the servos that drive the slider-crank mechanism, as well as sensors for monitoring vital metrics such as power consumption and internal temperature and humidity. We deploy a simple proportional control to map the angle of servo motor turn to the linear position of the potentiostat arm. The firmware on the microcontroller allows for modification of the speed of the servo motors and timestamps and saves the metrics from the sensors onto a microSD card for later use. The system diagram of different modules and how they interact with each other is shown in Fig. 3.

The mechanism of pressure/vacuum pulse generation is as follows. When the servo motor rotates, its rotational moment is converted to the reciprocating motion of the plunger or the arm of the air cylinder. The inherent construction of the air cylinder includes an air seal at the end of the plunger. Hence, as the plunger moves it compresses or expands the air trapped between one of its outlets and the microfluidic chip it feeds. The other outlet of the plunger serves as a vent throughput this operation.

**Fig. 1:**
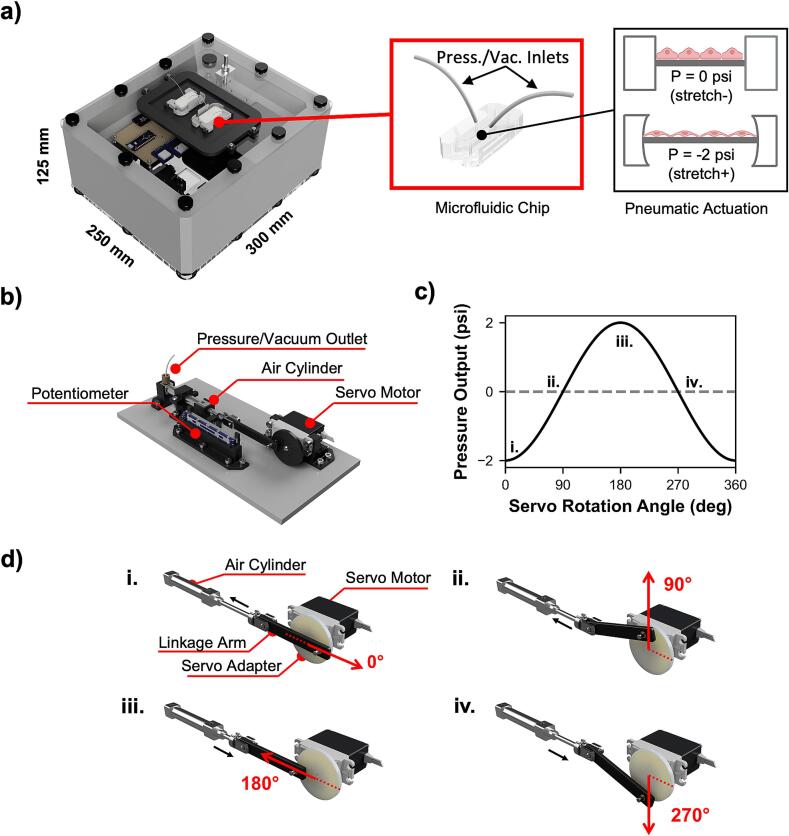
Overview of the proposed pulse generator. a) Render of assembled pressure controller box. The insets show an example of a microfluidic chip and the method of actuation of the chip under cyclic pressure and vacuum. b) Render of one of the two pressure/vacuum source units inside of the controller box. c) Example pressure output of the controller versus servo angle. The pressure generated by the cylinders oscillates between −2 and +2 psi within a single servo rotation. d) Renders of the linkage mechanism, showing servo rotation angles that correspond to i) minimum pressure (maximum vacuum), ii) and iv) neutral positions, and iii) maximum pressure. The numerals correspond to the labeled locations in the graph in c).

**Fig. 2:**
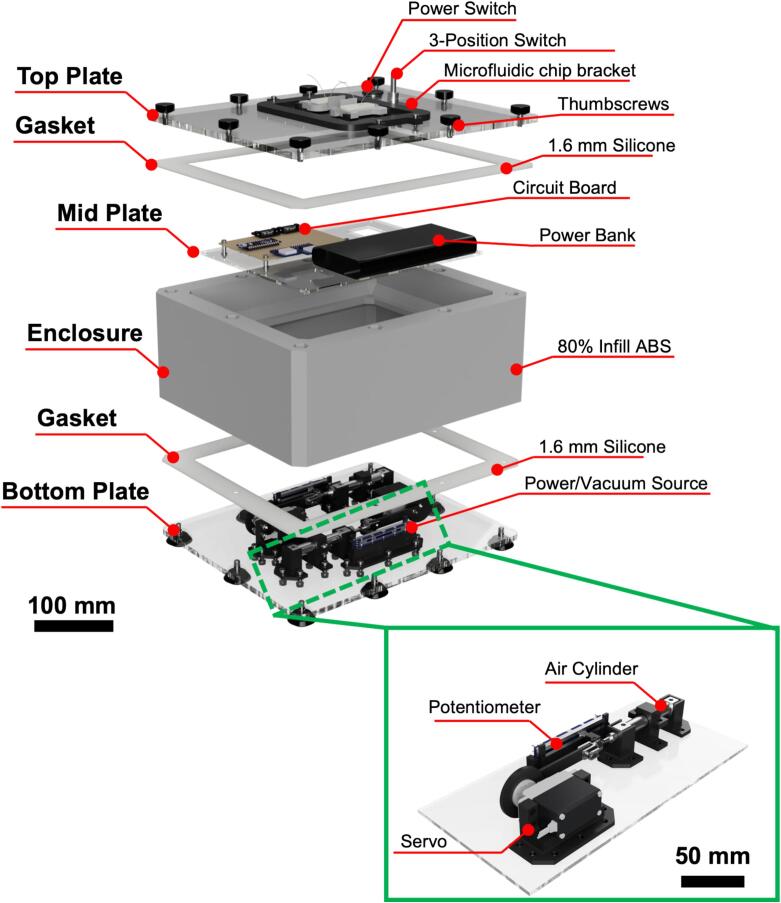
Exploded view of pressure controller showing the top, mid, and bottom plates as well as the 3D-printed enclosure, gaskets, and the slider-crank subassembly.

The proposed pressure controller is constructed using standard rapid prototyping techniques, including 3D-printing, laser cutting, and firmware development using Arduino microcontrollers. The controller unit is designed to be incubator-friendly and consists of a 300 × 250 × 125 mm (L × W × H) 3D-printed ABS enclosure, with dual cyclic pressure sources that can be used to actuate flexible microfluidic chips (Fig. 1a). The core of the pressure controller is the individual cyclic pressure source (Fig. 1b). The pressure source consists of a Parallax 360° continuous servo that drives a 30 mm pneumatic air cylinder through a custom 3D-printed linkage arm. The plunger of the air cylinder is also connected to a 10 kΩ linear potentiometer to allow for positional feedback and control. Although this model of Parallax servo can control relative position via its internal Hall effect sensor feedback loop, the potentiometer method allows for use with a wider variety of servo models and does not depend on the position of the plunger relative to the position of rotation of the servo shaft. The air cylinder can generate ±2 psi through roughly 12 in. of 1/16″ tubing, as measured during experimentation (Fig. 1c). A 0° angle of servo rotation corresponds to minimum pressure (maximum vacuum) of –2 psi (Fig. 1d i), while a 180° angle of servo rotation corresponds to maximum pressure (minimum vacuum) of 2 psi (Fig. 1d iii), and 90° and 270° angular rotations provide neutral (ambient) pressure (Fig. 1d ii, iv). Operating the servo at a continuous rotational speed provides a sinusoidal pressure/vacuum output that oscillates between these two extremes. The pressure controller is formed from several subassemblies, as seen in Fig. 2. The top plate (laser cut from 1/8″ acrylic) holds the user interface and a bracket for securing microfluidic chips during experiments. The user interface consists of a main power switch and a 3-position switch for controlling the movement of the air cylinders. The cylinders can be directed to hold at maximum pressure (switch in up position), to hold at minimum pressure (switch in down position), or to continuously cycle (switch in middle position). The top plate is attached to the enclosure with thumbscrews to allow ease of removal for power bank replacement or firmware adjustment. The mid plate (laser cut from 1/8″ acrylic) rests on a rim inside of the enclosure and allows for mounting of the controller circuit board and power bank. It also provides passthroughs for pneumatic tubing and electrical connections to the bottom plate. The bottom plate (laser cut from 1/4″ acrylic) holds all mechanical components that make up the dual pressure/vacuum sources of the controller. The bottom plate is secured to the enclosure using leveling feet. Attached to the bottom plate on the inside of the enclosure are the two of the pneumatic cylinder subassemblies that form the pressure/vacuum sources, each consisting of a continuous rotation servo motor, a potentiometer, and a pneumatic air cylinder. These components are mounted on custom designed 3D-printed mounts attached to the bottom plate via 14 mm M4 screws. The components are mechanically linked using further custom designed 3D-printed adapter wheels and linkage arms.

### 2.2 Electrical components

**Fig. 3:**
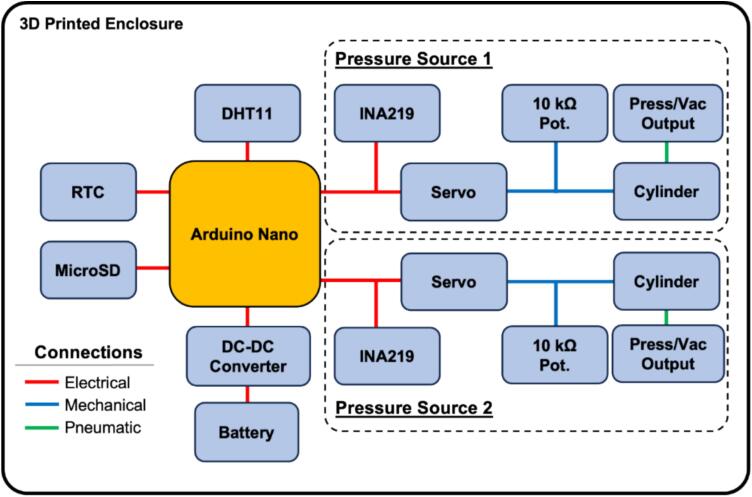
Block diagram of the pressure controller, showing electrical connections (red lines), mechanical connections (blue lines), and pneumatic connections (green lines). (For interpretation of the references to colour in this figure legend, the reader is referred to the web version of this article.)

A block diagram of the system is shown in Fig. 3, highlighting the connections (electrical, mechanical, and pneumatic) that branch out from the Arduino Nano that acts as the central control unit.

**Fig. 4:**
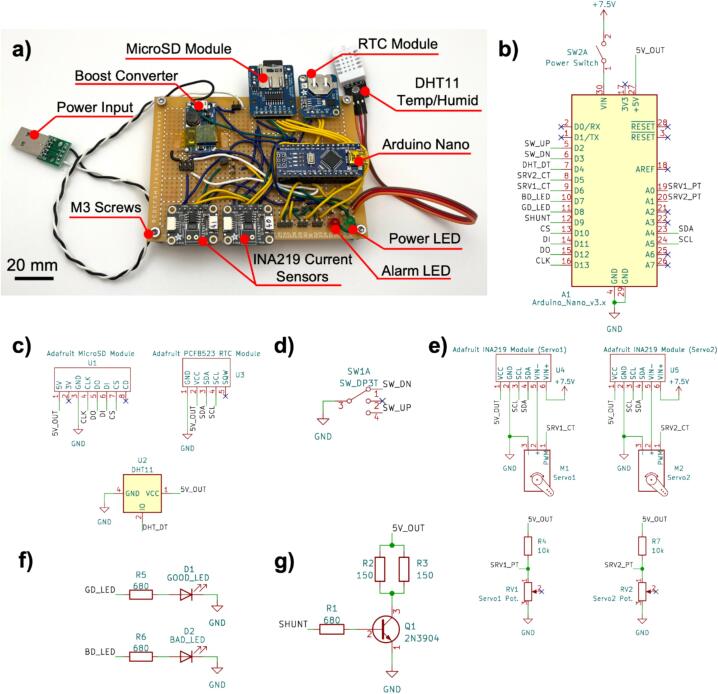
Arduino Nano powered controller circuit board. a) Picture of the assembled controller circuit board with labeled components. b) Schematic of the Arduino Nano microcontroller. c) Schematic of data logging modules and DHT11 temperature/humidity sensor. d) Schematic of three-position switch circuit for stroke control. e) Schematic of the servo control and feedback circuits. f) Schematic of LED indicator circuits. g) Schematic of power shunt circuit.

The servo motors of the pressure controller are operated via a custom battery-powered circuit board (Fig. 4a). An Arduino Nano microcontroller serves as the central component of the pressure control unit circuit, and the connections made to its pins are shown in Fig. 4b. A DHT11 temperature and humidity sensor unit and two Adafruit INA219 current sensor breakout boards (one for each servo motor) provide performance metrics over time (Fig. 4e). These metrics are timestamped with a date and time via an Adafruit PCF8523 real-time clock module and saved to a MicroSD card through an Adafruit 254 MicroSD card breakout board (Fig. 4c). The main reason for observing the current over time is to identify whether the controller failed at any point of time when inside the incubator. This could happen due to several reasons such as mechanical failure, tubing disconnects, electrical failure, running out of power and thus will be reflected in the current measured from the servo motor. The electronics for the pressure controller are powered by a 26,800 mAh power bank with a 5 V, 2.1 A output. The voltage to power the servos is boosted from 5 V to 7.5 V for increased torque using a DC-DC converter breakout board. The power bank has an overcharge protection circuit that shuts off the supply of power if the current drops below a particular value (in this case, ∼100 mA). When the servos are idling, as they do when sent to the maximum or minimum hold positions with the three-position switch (Fig. 4d), the current draw drops below 100 mA causing the power bank to shut off and the Arduino to reset. To avoid this issue, a power shunt circuit was added that drops 5 V across two 150 Ω resistors in parallel, leading to an additional ∼67 mA draw from the powerbank. This circuit is turned on or off via a bipolar junction transistor switch that is controlled through a digital output pin of the Arduino (Fig. 4g). LED indicators are included to display power status (green) and alarm status (red) (Fig. 4f). All components are soldered onto an 80 mm by 110 mm perfboard (Flinn Scientific #AP10014), and power is introduced from the power bank through a male USB connector. Headers can be used to attach the switches, servo motor power and control wires, and potentiometer connections to allow for ease of disassembly of the unit.

### 2.3 3D-printed components

**Fig. 5:**
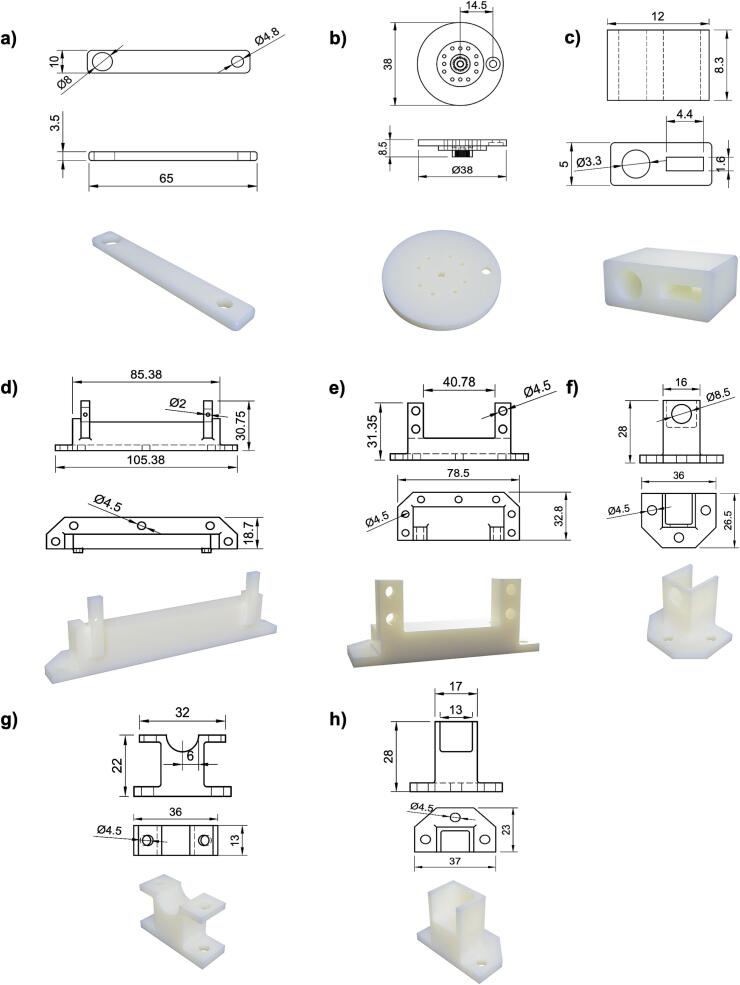
Drawings and renders of a) linkage arm, b) servo adapter, c) potentiometer linker, d) potentiometer support, e) servo motor support, f) servo front mount, g) servo mid mount, and h) servo rear mount. All dimensions are in mm.

Custom linkage components were designed to enable mechanical coupling of the rotational servo motion and the linear air cylinder motion via a slider-crank mechanism. Internal components were 3D printed using PLA, whereas the enclosure box was 3D printed using 80 % infill ABS to provide a strong barrier to water vapor. The linkage arm provided a smooth linear travel of the pneumatic cylinders (Fig. 5a, b). To couple the motion of the cylinder plunger with the linear potentiometer for positional feedback, a custom adapter was designed and printed(Fig. 5c). The rectangular slot in the adapter can be slid over the arm of the potentiometer, and ring lug can be heat-set into the circular slot which can be thread through the cylinder plunger so that the two components move in tandem. Mounts were designed and printed in PLA for attaching the potentiometer (Fig. 5d) and the servo motor (Fig. 5e) to the bottom plate. To support the air cylinder on the bottom plate, three mounts were designed and printed in PLA (Fig. 5f, g, h).

### 2.4 Laser cut plates

**Fig. 6:**
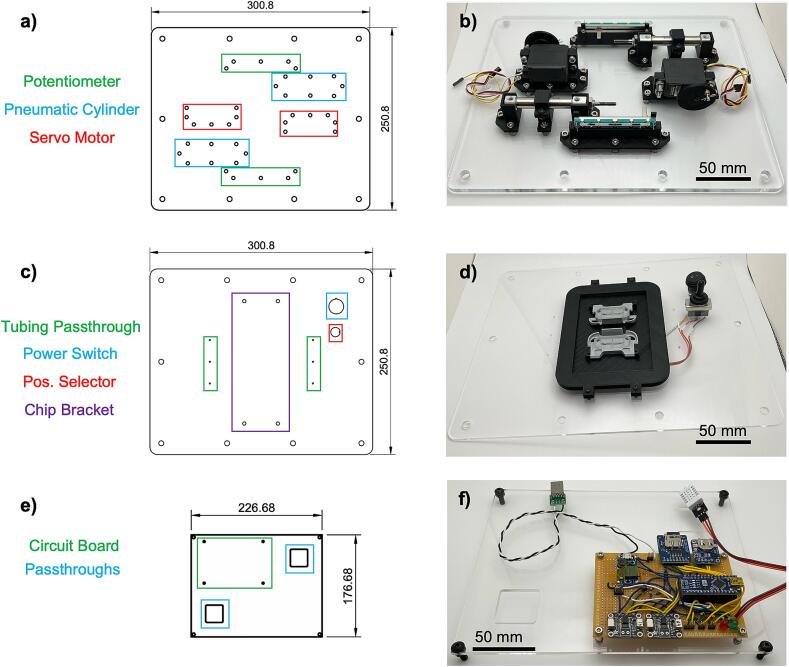
The internal levels of the box are fabricated from laser-cut acrylic. a) Drawing of base plate with locations of component mounts labeled, b) assembled base plate, c) drawing of top plate, d) assembled top plate, e) drawing of mid plate, f) assembled mid plate.

Components of the pressure controller are secured onto laser cut acrylic sheets, allowing for ease of manufacturing. The major goal of this design was to have a simple, user-friendly assembly of different components for prolonged operation. The bottom plate, holds the 3D-printed mounts for the potentiometers, air cylinders, and servo motors (Fig. 6a, b). It also includes holes for attaching to the enclosure. The top plate, holds the microfluidic chip bracket and the power and positional control switches, as well as small holes for passing through the pneumatic tubing (1/16″ OD, 1/32″ ID, FEP) attached to the air cylinders for output to the microfluidic chip (Fig. 6c, d). There are through holes to attach the top plate to the enclosure using thumbscrews. The mid plate, holds the circuit board and allows passthrough of pneumatic tubing and electrical wiring, and provides space for housing the power bank (Fig. 6e, f).

### 2.5 Firmware

A C++ library was created to provide an interface to the combined servo motor and potentiometer feedback system. This library can be implemented in Arduino sketches by first placing the library C++ and header files in the “libraries” folder inside the Arduino home directory and then including the header file at the top of the sketch, as with other third-party libraries. The library defines an Actuator class that tracks the digital input/output pin assignments for the servo control pin (output) and potentiometer feedback pin (input). The Actuator class includes methods for initializing the servo and controlling the servo position, as well as a utility function for converting a desired RPM to a pulse-width duration using the conversions outlined in the datasheet for the Parallax 360° High Speed Servo. The firmware that is loaded onto the microcontroller in the pressure control unit is in the form of an Arduino project file (.ino) and is editable using the standard Arduino IDE.

## 3 Design files summary

### 3.1 3D-printing design files

**Table:** 

**Design File Name**	**Design**	**File Type**	**Open Source License**
chip_bracket.step		STEP	CC BY 4.0
cylinder_front_mount.step		STEP	CC BY 4.0
cylinder_mid_mount_bot.step		STEP	CC BY 4.0
cylinder_mid_mount_top.step		STEP	CC BY 4.0
cylinder_rear_mount.step		STEP	CC BY 4.0
enclosure.step		STEP	CC BY 4.0
linkage_arm.step		STEP	CC BY 4.0
potentiometer_linker.step		STEP	CC BY 4.0
potentiometer_mount.step		STEP	CC BY 4.0
servo_adapter.setp		STEP	CC BY 4.0
servo_mount.step		STEP	CC BY 4.0

• *chip_bracket.step*: bracket for securing microfluidic chips to the top plate of the enclosure
• *cylinder_front_mount.step*: mount for supporting the front of the miniature air cylinder
• *cylinder_mid_mount_bot.step*: mount for supporting the middle of the miniature air cylinder
• *cylinder_mid_mount_top.step*: top of the middle mount for preventing vertical movement of the air cylinder during actuation
• *cylinder_rear_mount.step*: mount for supporting the rear of the miniature air cylinder
• *enclosure.step*: enclosure in which all components reside
• *linkage_arm.step*: arm for linking servo motor output to miniature air cylinder
• *potentiometer_linker.step*: linker for connecting arm of potentiometer to miniature air cylinder
• *potentiometer_mount.step*: mount for supporting 10 kΩ linear potentiometer
• *servo_adapter.step*: adapter for extending the diameter of the output shaft of the servo motor
• *servo_mount.step*: mount for supporting the servo motor

### 3.2 Laser cutter design files

**Table:** 

**Design File Name**	**File Type**	**Open Source License**	**Location**
bot_acrylic_plate.dxf	DXF	CC BY 4.0	
gasket.dxf	DXF	CC BY 4.0	
mid_acrylic_plate.dxf	DXF	CC BY 4.0	
top_acrylic_plate.dxf	DXF	CC BY 4.0

• *bot_acrylic_plate.dxf*: 1/4″ acrylic laser cut plate on which to mount miniature air cylinders, potentiometers, and servo motors. Also acts as the bottom opening of the enclosure
• *gasket.dxf*: 1/16″ silicone gasket for sealing top and bottom openings of the enclosure
• *mid_acrylic_plate.dxf*: 1/8″ acrylic laser cut plate on which to mount the circuit board and power bank
• *top_acrylic_plate.dxf*: 1/8″ acrylic laser cut plate on which to mount the user interface and chip bracket. Also acts as the top opening of the enclosure

### 3.3 Other design files

**Table:** 

**Design File Name**	**File Type**	**Open Source License**	**Location**
CylinderActuator.cpp	C++ source file	CC BY 4.0	
CylinderActuator.h	C++ header file	CC BY 4.0	
pressure_controller_firmware.ino	Arduino firmware file	CC BY 4.0	
pressure_controller.kicad_sch	KiCAD schematic file	CC BY 4.0	
BillOfMaterials.pdf	PDF file	CC BY 4.0	
BuildInstructions.pdf	PDF file	CC BY 4.0

• *CylinderActuator.cpp*: C++ source file for Cylinder Actuator library
• *CylinderActuator.h*: C++ header file for Cylinder Actuator library
• *pressure_controller_firmware.ino*: Arduino firmware file that is loaded onto the Arduino Nano microcontroller
• *pressure_controller.kicad_sch*: KiCAD schematic file that defines layout of electrical circuit
• *BillOfMaterials.pdf*: list of items needed to construct the controller, along with their prices and where to source them
• *BuildInstructions.pdf*: detailed instructions for how to build the pressure controller

## 4 Bill of materials summary

The bill of materials is available as a PDF document in the Mendeley Data repository-https://doi.org/10.17632/f8jzn29f4c.2.

## 5 Build instructions

### 5.1 3D-printing and laser cutting

1. 3D-print the enclosure (enclosure.step) using 80 % infill ABS using a 0.2 mm nozzle at 230 °C and a bed temperature of 100 °C
2. 3D-print all other components using 20 % infill PLA using a 0.2 mm nozzle at 215 °C and a bed temperature of 60 °C
  a. chip_bracket.step
  b. cylinder_front_mount.step
  c. cylinder_mid_mount_bot.step
  d. cylinder_mid_mount_top.step
  e. cylinder_rear_mount.step
  f. linkage_arm.step
  g. potentiometer_linker.step
  h. potentiometer_mount.step
  i. servo_adapter.step
  j. servo_mount.step
3. Laser cut base plate pattern (bot_acrylic_plate.dxf) out of ¼” thick acrylic (laser power: 45 W, speed: 15 mm/s, 4 passes)
4. Laser cut top plate (top_acrylic_plate.dxf) and mid plate (mid_acrylic_plate.dxf) patterns out of 1/8″ acrylic (laser power: 45 W, speed: 15 mm/s, 2 passes)

### 5.2 Assembling small internal components

5. Linkage arm (linkage_arm.step) (Fig. 5a)
  a. This linkage arm connects the rotation output of the servo to the pneumatic cylinder to allow for cyclic actuation of the cylinder
  b. Press-fit 1/8″ inner diameter oil-embedded sleeve bearing (McMaster-Carr #6391K611) into the smaller hole of linkage arm using a vise
  c. Press-fit 4 mm inner diameter oil-embedded sleeve bearing (McMaster-Carr #6658K114) into the larger hole of the linkage arm using the same method as in (b) above
6. Servo adapter (servo_adapter.step) (Fig. 5b)
  a. The servo adapter is a circular chuck that fits onto the Parallax 360° Continuous Servo to extend its radius so that the full range of the 30 mm cylinder plunger can be actuated. It contains a hole for attaching a Clevis pin which will connect to the previously assembled linkage arm
  b. Insert the 1/8″ diameter Clevis pin (McMaster-Carr #98306A611) into the servo adapter using a vise
  c. Attach the servo adapter to the Parallax 360° Continuous Servo by pressing it onto the spline and securing it with a 12 mm M3 screw (McMaster-Carr #92095A183)
  d. Add a 1/8″ inner diameter oil-embedded sleeve bearing (McMaster-Carr #6391K611) to the Clevis pin to allow the linkage arm to remain parallel to the travel of the pneumatic cylinder
7. Potentiometer-cylinder linker (potentiometer_linker.step) (Fig. 5c)
  a. This linker connects the plunger rod of the cylinder to the 10 kΩ potentiometer to allow for detection of position. The component has a circular hole for inserting a lug that will attach to the cylinder plunger rod, and a rectangular hole that slips over the shaft of the linear potentiometer
  b. Remove the plastic portion of the M4 ring connector lug (DigiKey #WM18274CT-ND)
  c. Position the ring connector lug in the circular hole on the 3D-printed component with the ring facing toward the rectangular hole and heat-set it using a soldering iron set to 250 °C

### 5.3 Assembling internal components

8. The baseplate of the pressure control unit consists of a laser cut 1/4″ thick acrylic sheet with holes for attaching the 3D-printed component mounts and for attaching the plate to the main enclosure body (Fig. 6a)
9. All component mounts are attached to the baseplate using 14 mm M4 screws (McMaster-Carr #91292A038), with washers (McMaster-Carr #98689A113), lock washers (McMaster-Carr #94241A520), and nuts (McMaster-Carr #91828A231) to prevent vibration-induced failure (Fig. 6b)
10. Pneumatic cylinder mounts (Fig. 5f, g, h)
  a. Attach the rear cylinder mount (cylinder_rear_mount.step) to the baseplate using x3 14 mm M4 screws
  b. Attach the middle cylinder mount (cylinder_mid_mount_bot.step) to the baseplate using x2 14 mm M4 screws
  c. Attach the front cylinder mount (cylinder_front_mount.step) to the baseplate using x3 14 mm M4 screws
  d. Pass the plunger side of the pneumatic cylinder through the hole in the front mount and secure it with x1 M8 nut (McMaster-Carr #90710A120)
  e. Attach the middle top mount (cylinder_mid_mount_top.step) to the middle cylinder mount with x2 12 mm M4 screws (McMaster-Carr #92095A192) to prevent vertical motion
11. Potentiometer mounts (potentiometer_mount.step) (Fig. 5d)
  a. Install the mounts onto the baseplate with x5 14 mm M4 screws
  b. Install the potentiometers onto the mounts using x2 4 mm M2 screws (McMaster-Carr #90116A008) with M2 washers (McMaster-Carr #93475A195) and lock washers (McMaster-Carr #92148A050)
12. Servo motor mounts (servo_mount.step) (Fig. 5e)
  a. Install the mounts onto the baseplate with x2 14 mm M4 screws
  b. Install the servos onto the mounts using 20 mm M4 screws (McMaster-Carr #91292A121) with washers, lock washers, and nuts
13. Linking the pneumatic cylinder plunger, potentiometer, and servo motor
  a. Slide the rectangular hole of the previously assembled potentiometer linker onto the arm of the potentiometer (Fig. 7a)

**Fig. 7:**
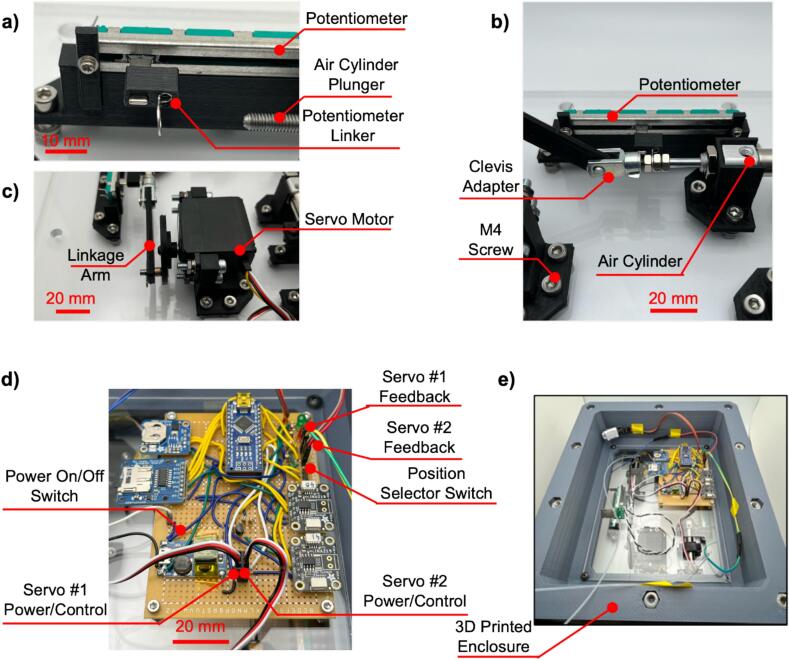
Slider-crank mechanism and circuit board header installation details. a) Installation of potentiometer – cylinder adapter onto potentiometer arm, b) adapter secured between M4 nuts on pneumatic cylinder plunger, linkage arm connected with Clevis rod adapter, c) other end of linkage arm connected to servo adapter, d) close-up of circuit board with labeled header connections, e) mid plate attached to 3D-printed enclosure.
  b. Attach x2 3.2 mm M4 nuts (McMaster-Carr #91828A231) all the way onto the threaded plunger of the pneumatic cylinder, pass the plunger through the eye lug of the potentiometer linker, and secure with a third M4 nut on the other side
  c. Pass a Clevis rod adapter with an M4 thread (McMaster-Carr #2448K41) through the larger oil embedded sleeve bearing on the previously assembled linkage arm
  d. Thread the Clevis rod adapter onto the end of the cylinder plunger to attach the linkage arm to the pneumatic cylinder (Fig. 7b)
  e. Attach the opposite end of the linkage arm to the Clevis pin on the servo adapter and secure it in place with a Cotter pin (McMaster-Carr #92375A610) through the eye hole of the Clevis pin (Fig. 7c)
14. Use 10–32 pneumatic adapters (Cole-Parmer #EW-02020-55) to attach 1/16″ outer diameter tubing (VWR # MFLX06406-60) to the pneumatic cylinders

### 5.4 Assembling the circuit board and installing the firmware

15. The components can be hand-soldered onto an 80×110 mm perfboard (Flinn Scientific #AP10014) in the configuration dictated by the schematics in Fig. 4. An example of a suggested component layout can be seen in Fig. 4a
16. The DC-DC boost converter has an output voltage range of 5 V – 28 V and should be set to 7.5 V to allow for more torque to be available to the servo motors. This can be accomplished by adjusting the on-board potentiometer while measuring the output voltage
17. One of the two INA219 current sensors must have its I2C address changed to 0x41 from the default 0x40 to allow for simultaneous communication of both sensors with the Arduino. This can be accomplished by shorting the A0 jumper on the back of the board
18. In order to enable easy disassembly for any potential maintenance, it is recommended that 2.54 mm pitch headers are soldered onto the board for servo control and communication wires (3 pins, x2), the power input (2 pins, x1), the mode selection switch (3 pins, x1), and the potentiometer monitoring wires (2 pins, x2)
19. Ensure that the real time clock has been programmed with the correct date and time
20. Ensure that there is a MicroSD card in the MicroSD card reader
21. To compile and use the Arduino.ino sketch file, it is necessary to place the custom Arduino library (CylinderActuator) in the “Arduino/libraries” folder of your filesystem, as is typically done for third-party libraries
22. Make any desired edits to the firmware, and then load the firmware onto the Arduino Nano via USB

### 5.5 Assembling the top and mid plates

23. Secure the 3D-printed chip bracket (chip_bracket.step) to the top plate using x4 16 mm M3 screws (McMaster-Carr #91292A115) secured with M3 washers (McMaster-Carr #93475A210), lock washers (McMaster-Carr #92148A150), and nuts (McMaster-Carr #91828A211) (Fig. 6d)
24. Attach the circuit board to the mid plate via x4 16 mm M3 screws (McMaster-Carr #91292A115) with 6 mm spacers (McMaster-Carr #94669A101), and secured with M3 washers (McMaster-Carr #93475A210), lock washers (McMaster-Carr #92148A150), and nuts (McMaster-Carr #91828A211) (Fig. 6f)
25. Press-fit the power switch into its corresponding hole
26. Attach the position selector switch into its hole and attach it with its included hardware

### 5.6 Assembling the enclosure

27. Install x20 ¼”-20 hex nuts (McMaster-Carr #97619A890) in the hexagonal recesses located around the top (x10) and bottom (x10) of the 3D-printed enclosure, and secure them in place using super glue
28. Attach the previously assembled base plate (with servo motors, pneumatic cylinders, and potentiometers installed) onto the enclosure and secure it in place using x10 ½” long ¼”-20 leveling feet (McMaster-Carr #2284T51)
29. Pass the servo motor power, servo motor control, and potentiometer feedback wires up from the base plate through the rectangular passthroughs in the mid plate, and attach them to their respective headers on the circuit board (Fig. 7d)
30. Attach the mid plate to the middle shelf of the enclosure using x4 16 mm M5 screws (McMaster-Carr #91292A126) and x4 M5 nuts (McMaster-Carr #91828A241) (Fig. 7e)
31. Pass the pneumatic tubing from the output of the pneumatic cylinders through the same rectangular passthroughs
32. Attach the power bank to the circuit board using the USB adapter
33. Hold the top plate above the enclosure and attach the power and position selector switch wires to their respective headers on the circuit board
34. Pass the pneumatic tubing from the pneumatic cylinders through the small holes in the top plate adjacent to the chip bracket
35. Secure the top plate to the enclosure using ½” long ¼”-20 thumbscrews (McMaster-Carr #91185A398)
36. A thin silicone gasket can be included between the enclosure and the top and bottom plates for an increased moisture barrier

## 6 Operation instructions

Operation of the pressure control unit is intended to be very simple once assembly is complete. The first step is to connect the output tubing of the unit to your application. Then, simply turn the unit on using the power switch. For continuous cyclic pressure and vacuum, ensure that the mode selector switch is in the middle position. For maximum plunger depression (and thus maximum positive pressure), move the switch to the “up” position. For maximum plunger extension (and thus maximum vacuum), move the switch to the “down” position. A fully charged power bank lasts for ∼3.5 days of continuous usage and can be swapped by simply removing the top thumbscrews and attaching a freshly charged power bank to the USB adapter. The frequency of the cyclic pressure and vacuum can be adjusted by modifying the STEADY_RPM variable in the firmware and uploading the new version to the microcontroller.

## 7 Validation and characterization

**Fig. 8:**
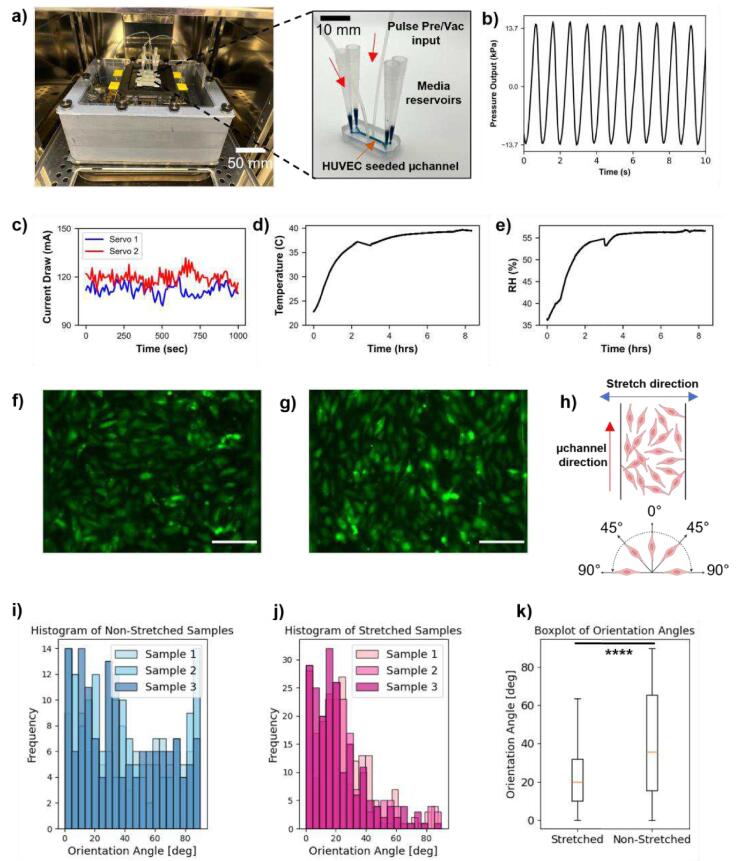
Experimental results. a) Assembled control box with microfluidic chips attached inside of an incubator ready for an experiment, b) pressure versus time output, showing frequency of ∼1 Hz, c) current draw of servos 1 and 2 versus time, showing a consistent current draw of ∼115–120 mA, d) internal temperature, and e) internal relative humidity (RH) of box over the duration of the 8-hour experiment, f) fluorescent imaging of GFP + HUVECs in the absence of cyclic stretch, g) fluorescent imaging of GFP + HUVECs subjected to cyclic stretch over 8 h, h) diagram of cell orientation in the microfluidic channel, i) and j) are showing histogram of cell orientation relative to vertical of non-stretched cells and stretched cells respectively. k) Box plot showing statistical significance with p < 0.001 between the experimental control and the treatment group. Scale bars in f) and g) are 100 µm.

To validate the ability of the controller to actuate a microfluidic stretch channel, a cell stretch assay was performed using green fluorescent protein expressing human umbilical vein endothelial cells (GFP + HUVECs, Neuromics #GF01) in Emulate Inc Chip-S1™ chips. This chip has two microchannels on top of each other, separated by a porous PDMS membrane. The top channel cross sectional area is 1mmx1mm where HUVECs are seeded. The bottom microchannel dimensions are 1mmx0.2 mm (Fig. 8a). This channel was primed with media prior to the stretching experiment. The membrane pore diameter is 7 µm and the thickness is 50 µm. One of the primary reasons for using this commercial chip was it is well validated, and the design is optimized to generate uniform stretching across the membrane. HUVECs are frequently used as a model for the response of endothelial cells to shear stress. They can show alignment perpendicular to the axis of uniaxial cyclic stretch in as little as 60 min, which can easily be observed using fluorescence microscopy.

The stimulation parameters, i.e. pressure and frequency, are different for different cell types. Our current estimates were based on the published literature in the Table 1 as well as the Emulate’s chip operations manual which states the maximum pressure should not exceed 35 psi. We would like to point that ±2 psi is the pressure measured using a pressure sensor (Adafruit MPRLS Ported Pressure Sensor Breakout – 0 to 25 PSI Product ID: 3965) which had almost 6 times the dead volume as that of the chip. Thus, we anticipate that the actual pressure on the membrane must be higher that the measured value. Using ideal gas laws, simple calculation would yield this value to be around 8 psi (after subtracting the tubing dead volume). Final validation of this value was confirmed by the positive cell stretch assay results. For users interested to generate higher ranges of pressures/vacuum, the current air mini cylinder can be substituted with a larger capacity one.

**Table 1:** List of previously published cell-stretch devices compatible with the battery powered pressure controller.

Sr. No.	Device and elastic substrate material	Peripheral equipment and control system	Stimulation parameters	Application/in-vitro model system	Reference
1.	PDMS	Vacuum pump, solenoid valve, PC	2.3 Hz (80 bps)	Artery smooth muscle cells	K. Sato, et.al.[17]
2.	PDMS	Programmable pressure controller,	1 Hz cyclic Pressure	primary bovine aortic endothelial cells	P. Chu, et.al.[18]
3.	PDMS	Pressure controller (Fluigent), Vacuum Pump, Nitrogen tank, PC	0–1 Hz	HUVECs and hMSCs in 2D and Matrigel	Y. He, et.al. [19]
4.	PDMS	Programmable air pump (Elveflow)	50 mbar at 0.5 Hz	primary alveolar epithelial type 2 (AT2) cells	V. Kumar, et.al.[20]
5.	PDMS	Vacuum regulator (ITV0091-2BL, SMC Corporation of America) controlled using Arduino Leonardo and MAX517 digital to analogue converter	1 Hz, 85 kPa	Kidney-on-chip	S. Musah, et.al.[21]

The GFP + HUVECs were cultured for 6–7 days in gelatin-coated (Millipore #ES-006) tissue culture flasks in EBM™-2 Endothelial Cell Growth Basal Medium-2 (Lonza #CC-3156) supplemented with EGM™-2 SingleQuots™ Supplements (Lonza #CC-4176), 100 U/mL Penicillin/Streptomycin (Thermo Fisher Scientific #15140-122), 50 mg Normocin (Invitrogen #ant-nr-2), and 10 % FBS (Lonza #CC-4176). The cells were loaded into the chips at a concentration of 1.8 × 10^5^ cells/mL, passed through the chip and allowed to settle for 30 min. This process was repeated twice, and the cells remaining in the chip were left overnight to allow for adhesion to the channel.

The chips were attached to the enclosure using the 3D-printed chip bracket, and the pressure output of each pneumatic cylinder was split into two lines using a Y-splitter (Cole-Parmer #EW-02024-07) before being fed into the two input ports on each chip. The assembled box with chips attached was placed in an incubator (Fig. 8a) and subjected to a pressure and vacuum cycle of +2 psi / −2 psi at a frequency of ∼1 Hz (Fig. 8b) for 8 h total. The limit to the frequency of the pneumatic signal is dictated by the rpm of the servo as well as compression dynamics of the plunger inside the pneumatic cylinder. The reason we chose this frequency was because it was like what has been deployed in the literature in similar studies. We have attached a table, Table 1, that list similar devices and stimulation parameters. A frequency lower or higher than 0.5 Hz is totally feasible by modulating the input PWM control signal and voltage supplied to the servo motor. Our choice of 8hr. long stimulation of the HUVEC cells was based on several previously published studies. As an example, Yoshigi et.al.[22] quantified cytoskeletal remodeling of human endothelial cells under cyclic stretching. They observed that in the case of **confluent** HUVECs, between 2–20 hrs. of stimulus at 0.5 Hz was enough to cause change in the orientation with a peak approximately at 5hrs. Similar studies on endothelial and epithelial cells were reported by Wang et.al.[23] who showed through phase contrast microscopy and histogram analysis, large population of cells reoriented only after 3hrs of cyclic mechanical stretching. Additional studies have also been reported with mesenchymal stem cells in microfluidic cell stretch chips for similar duration of stimulation [24], [25]. Given these published results, we chose our initial cell seeding density such that HUVECs formed confluent layer throughout the span of the microchannel, before the application of cyclic stretching. This ensured that an acute exposure of 8hrs was sufficient to reorient them perpendicular to the stretch's direction. An additional two chips containing GFP + HUVECs were kept static for a control experiment in the same incubator. Post-experiment fluorescent microscopy showed alignment of cells perpendicular to the stretching direction for the stretched cells, while the non-stretched cells remained without preferential alignment (Fig. 8f, g). Analysis of the orientation of each cell in the images was conducted using a custom Python script (see *angle_analysis.py* and the corresponding procedure *StretchAnalysisProcedure.pdf* in the supporting information repository).Fig. 8 (i) and (j) shows the histogram for cellular orientation angles plotted for three different samples in non-stretched and stretch conditions respectively. Fig. 8 (k) shows the box plot summarizing the distributions of the treatment group (stretched) and control group (non-stretched). An independent *t*-test was performed in PYTHON 3 which resulted in the p-value of 1.25e-10 showing that the results obtained are statistically significant. The output of the cell orientation python program as well as statistical analysis programs are provided in the supporting information repository.

## 8 Conclusion and future work

Cell stretching is an important facet of homeostasis since cells are constantly exposed to such stimulus. Consequently, several organ-on-chip devices have incorporated cyclic cell stretch functionality to model these effects in-vitro. Our primary choice for using Emulate chips was that it is the current gold standard in organ-on-chip devices and several model systems have been deployed on similar architecture such as lung-on-chip, gut-on-chip, kidney-on-chip. However, other devices with custom brackets can be easily deployed with this controller. Table 1**.** enlists studies that are published in the literature with which our pressure controller will be directly compatible with in a plug-and-play manner. All these devices, otherwise need bulky apparatus, such as air compressors, solenoid valves, PC with control software, to generate similar pneumatic stimulation as reported in the experimental sections of these papers.

Several modifications to the current design could be made to help improve usability. An off-the-shelf power bank was used to power the system for this study and was suboptimal since it required an additional transistor circuit to continuously draw power to avoid auto shutoff. However, one could also use a rechargeable battery with a recharging circuit which would not be prone to automatic shutoffs and eliminate the need for the transistor circuit. Likewise, USB feedthroughs can be added to the enclosure to allow for easier access to battery charging and Arduino programming. One point to note is that this port must be fitted with a circular gasket so there isn’t any leakage around the cable to ensure reliable operation in the incubator. We used PLA for internal fixtures and 80 % infill ABS for the enclosure. However, several other prototyping materials and 3D printing methods can be used if they are mechanically strong and show good resistance with humidity.

One of our primary goals for this device is to assess the integrity of the HUVEC barrier over several days of exposure to various chemotherapy drugs, which requires sustained operation in the incubator's humid environment. We would like to highlight that in our 8-hour experiments, the system exhibited no signs of fatigue failure, whether mechanical or electrical/electronic. The choice of this shorter duration was based on published studies, not due to any inherent limitations of the system. Temperature and humidity levels stabilized during these tests, suggesting the system’s potential to function beyond 8 h. Nonetheless, we are continuing to evaluate potential long-term issues, such as corrosion from prolonged humidity, high-temperature spikes, and mechanical failures. The findings from these long-term studies, along with their application to more extensive drug testing protocols, will be detailed in our next publication. This ongoing work will provide valuable insights into the system's long-term performance and its potential applications in sustained drug exposure studies. Additionally, since strain characteristics depend on the membrane characteristics and device geometry, we have only included gauge pressure output of the controller. In case of the Emulate chips, which were used in the current study, 2 psi was within the prescribed range. However, we didn’t measure this strain directly and we currently lack the appropriate apparatus to evaluate this parameter. For the future studies, we will include this characterization.

## 9 Ethics statements

No human or animal subjects were involved in the presented work.

## CRediT authorship contribution statement

**Samuel Olson:** Writing – original draft, Visualization, Validation, Software, Investigation, Data curation, Conceptualization. **McKenna Finley:** Writing – review & editing, Validation. **Raviraj Thakur:** Writing – review & editing, Visualization, Supervision, Project administration, Methodology, Funding acquisition, Conceptualization.

## Declaration of competing interest

The authors declare that they have no known competing financial interests or personal relationships that could have appeared to influence the work reported in this paper.
